# Antifungal Susceptibility Trends Among Filamentous Fungi: An Epidemiological Evaluation on *Aspergillus* spp., *Fusarium* spp., and *Scedosporium* spp. from Southern Italy

**DOI:** 10.3390/antibiotics15020146

**Published:** 2026-02-02

**Authors:** Maddalena Calvo, Marta Caccamo, Dalila Maria Cammarata, Laura Trovato

**Affiliations:** 1 U.O.C. Laboratory Analysis Unit, A.O.U. Policlinico “G. Rodolico-San Marco” Catania, 95123 Catania, Italy; maddalenacalvo@gmail.com; 2 Department of Biomedical and Biotechnological Sciences, University of Catania, 95123 Catania, Italy; caccamomarta@gmail.com (M.C.); dalilacammarata@gmail.com (D.M.C.)

**Keywords:** *Aspergillus* spp., *Scedosporium* spp., *Fusarium* spp., antifungal resistance, antifungal susceptibility testing, epidemiology

## Abstract

**Background/Objectives**: Antifungal resistance among filamentous fungi is an increasing global concern with significant implications for clinical management. Herein, we propose a study aiming to investigate in vitro susceptibility patterns and epidemiology of filamentous fungi in Southern Italy, focusing on MIC distributions and resistance trends. **Methods**: We reported susceptibility results from *Aspergillus* spp., *Fusarium* spp., and *Scedosporium*/*Lomentospora* spp. clinical isolates, which underwent azoles, echinocandins, and amphotericin B in vitro testing. **Results**: *Aspergillus fumigatus* was the most frequently isolated species, showing an alarming increase in reduced susceptibility to amphotericin B (9.1%). The highest MIC ranges for this antifungal drug emerged in the case of *A. fumigatus* (1–4 mg/L) and *A. terreus* (2–8 mg/L), while *A. flavus* (0.5–4 mg/L) and *A. niger* (0.25–4 mg/L) showed lower values. As regarding azoles, all the *Aspergillus* spp. strains exhibited variable MIC values, reporting a 0.06–16 mg/L MIC range for itraconazole, 0.125–1 mg/L for voriconazole, and 0.03–1 mg/L for posaconazole. *Fusarium solani* exhibited high MICs for azoles (8 mg/L) and amphotericin B (2–4 mg/L), while *F. oxysporum* and *F. proliferatum* showed lower MICs (0.25–2 mg/L for amphotericin B and a MIC range of 0.5–8 mg/L for posaconazole). *Lomentospora prolificans* and *Scedosporium apiospermum* demonstrated multidrug resistance across all tested antifungals, reporting MIC ranges of 4–8 mg/L for amphotericin B, 0.25–16 mg/L for posaconazole, 0.25–8 mg/L for voriconazole, and 0.125–8 for itraconazole. **Conclusions**: Our data highlight the critical emergence of reduced antifungal susceptibility among filamentous fungi in Southern Italy, underlining the importance of epidemiological surveillance, precise species identification, and optimized susceptibility testing in the case of mould etiology for invasive fungal infections.

## 1. Introduction

Invasive mould infections affect blood vessels, deep tissues, and organs after conidia inhalation or traumatic insertion. Respiratory transmission and cut or wound contaminations through fungal fragments correlate with mould infections [1]. Aspergillosis is one of the most common invasive mould infections, expressing several clinical forms, such as pulmonary aspergillosis, cerebral aspergillosis, and osteomyelitis, along with rare invasive conditions, such as endocarditis and gastrointestinal aspergillosis [2]. The World Health Organization’s latest report on alert isolates described *Aspergillus fumigatus* as the main clinically relevant species, counting it within the critical group of the priority list [3]. However, *Aspergillus flavus*, *Aspergillus terreus*, and *Aspergillus niger* significantly contribute to human pathogenesis, especially regarding respiratory infections [4].

Invasive pulmonary aspergillosis (IPA) expresses a consistent impact on patients reporting specific risk factors such as neutropenia, solid organ or stem-cell transplants, immunological impairments, corticosteroids therapies, mechanical ventilation, and chronic respiratory diseases [2,4]. As regards other severe fungal infections, invasive *Fusarium* spp. infections affect either immunocompetent or immunocompromised patients. Specifically, patients with leukemia or undergoing bone marrow transplants are possibly affected by dissemination episodes. On the other hand, immunocompetent patients may be injured by localized diseases such as keratitis [5]. The *Fusarium solani* complex (*F. solani sensu stricto*, *F. keratoplasticum*, *F. falciforme*) includes the most important human fusariosis aetiological agents, causing both bloodstream infections and keratitis [5,6,7]. Furthermore, *Fusarium oxysporum* has been isolated from keratitis and is invasive, while *Fusarium proliferatum* is an uncommon human pathogen rarely isolated in the case of respiratory infections [5,6,7,8]. According to these assumptions, the WHO integrated *Fusarium* spp. within the high-priority fungal pathogens [3]. *Scedosporium* spp. integrate the panel of possible rare mould aetiologies, including *Scedosporium apiospermum* complex (*Scedosporium apiospermum sensu strictu*, *Scedosporium boydii*) and *Lomentospora prolificans* [9,10]. All of these species colonize human respiratory airways, occasionally causing invasive infections among immunocompromised patients [10]. Moreover, *Scedosporium* spp. have been indicated as a frequent colonizing agent of cystic fibrosis patients’ respiratory tract [11]. The WHO guidelines included both *Scedosporium apiospermum* and *Lomentospora prolificans* among medium-priority fungal pathogens [3].

Despite difficulties in filamentous fungi management within the laboratory diagnostic routine, several methodologies have been applied for in vitro susceptibility testing. Clinical microbiology workflows mainly include gradient tests and broth microdilution [12,13]. The Clinical and Laboratory Standards Institute (CLSI) standardized performance instructions about filamentous fungi susceptibility testing [14], reporting clinical breakpoints and/or epidemiological cut-off values only for certain antifungal drugs against *Aspergillus* spp. [15]. The European Committee on Antimicrobial Susceptibility Testing (EUCAST) established comparable guidelines for *Aspergillus* spp. [16,17]. Unfortunately, all the above-mentioned documents do not include reference for *Fusarium* spp. or *Scedosporium* spp., except for quality control CLSI references [14,15].

Experimental studies highlighted some specific susceptibility patterns for the clinically relevant filamentous fungi. *Aspergillus* spp. reported azole resistance due to *cyp51A* gene mutations, which may alter the targeted 14-α-sterol demethylases. Membrane permeability variations and efflux pump overexpression contribute to the same resistance profile, which has been extensively related to environmental isolates and their exposure to fungicide azole agriculture usage. Echinocandin resistance may be related to 1,3-β-d-glucan synthetase gene mutations, even if these molecules are occasionally used because of their fungistatic activity against three spp. [18].

*Fusarium* spp. frequently exhibit intrinsic resistance to azoles, partly attributable to the reduced binding affinity of 14-α-sterol demethylase. By contrast, amphotericin B resistance is linked to changes in ergosterol content or composition of the fungal cell membrane and enhanced stress response mechanisms.

*F. solani* shows the most concerning susceptibility pattern, often including high amphotericin B MIC values and intrinsic azole resistance. Furthermore, echinocandins are not an appropriate treatment option in the case of fusariosis, due to the capability of *Fusarium* spp. to express 1,3-β-d-glucan synthetase gene mutations [19]. A multi-drug intrinsic resistance to azoles and amphotericin B characterizes *Scedosporium* spp. and *L. prolificans*. *Scedosporium* spp. reveal ergosterol modifications or efflux pump overexpression, while *L. prolificans* also produces hydrolytic enzymes able to inactivate antifungal drug structure. The previously cited echinocandin resistance mechanisms are constitutively present within *Scedosporium* spp. and *L. prolificans* [20]. Since several published studies have documented an emerging resistance phenomenon for filamentous fungi [21,22], it is essential to enrich the scientific literature about their susceptibility profiles. Herein we present an epidemiological analysis of *Aspergillus* spp., *Fusarium* spp., and *Scedosporium* spp., aiming to describe their in vitro antifungal susceptibility trends in Southern Italy.

## 2. Results

**Figure 1:**
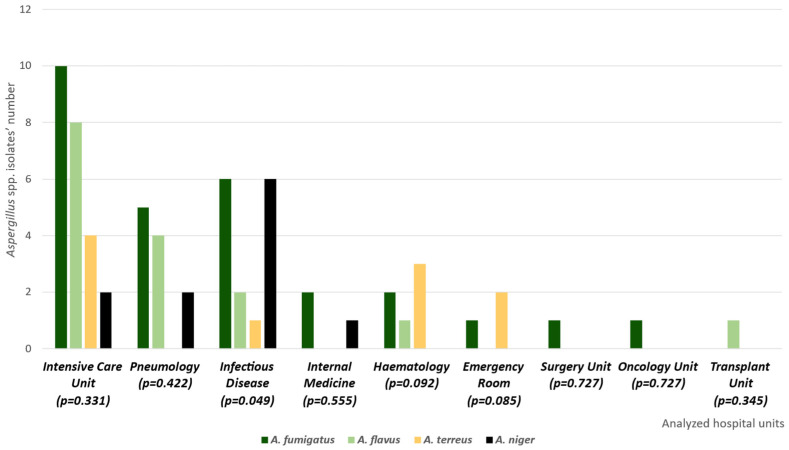
Distribution of *Aspergillus* spp. isolates within the analyzed hospital units. The graph illustrates the different *Aspergillus* species’ distributions, also describing the eventual statistical significance (*p* < 0.05) between the identified species and the analyzed hospital ward.

A total of 65 *Aspergillus* spp. isolates were collected during the study period. Among the 28 *A. fumigatus* strains, 13 emerged from bronchoalveolar lavage fluids (BALs), 5 from bronchial aspirates, and 10 from sputum samples. Eleven *A. flavus* samples were derived from bronchial aspirates, four from sputum samples, and one from BAL, globally accounting for sixteen strains. The analysis also included 11 *A. niger* isolates, isolated from BAL (6) and bronchial aspirates (5). We isolated 10 *A. terreus* samples from bronchial aspirates (4), BAL (3), and sputum samples (3). These respiratory isolates allowed an infection classification (probable pulmonary aspergillosis according to the European Organization for Research and Treatment of Cancer and the Mycoses Study Group Education and Research Consortium (EORTC/MSGERC) guidelines) [23,24]. Figure 1 illustrates the *Aspergillus* spp. isolate distribution within the included hospital units, along with a statistical analysis of this diffusion. This analysis demonstrates the statistical significance of the *Aspergillus* spp. distribution within the infectious disease unit. Furthermore, Table 1 summarizes antifungal susceptibility details for the same strains.

**Figure 2:**
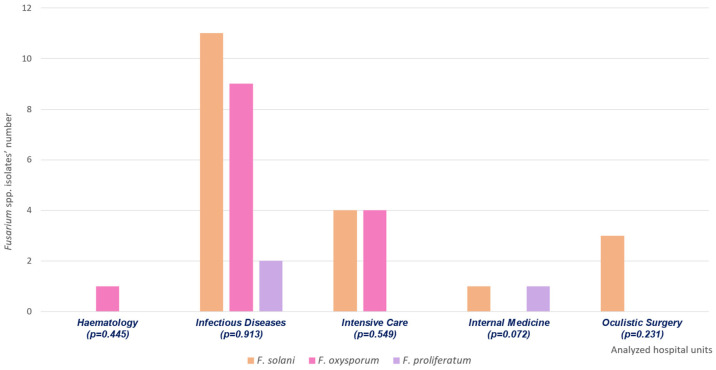
Distribution of *Fusarium* spp. isolates within the analyzed hospital units. The graph illustrates the different *Fusarium* species’ distributions, also describing the eventual statistical significance (*p* < 0.05) between the identified species and the analyzed hospital ward.

The study included 36 *Fusarium* spp. isolates, including 19 *F. solani*, 14 *F. oxysporum*, and 3 *F. proliferatum*. Figure 2 shows their distributions within different hospital units, along with a statistical analysis of the eventual significance of species distributions in specific wards.

Regarding *F. solani*, eleven isolates emerged from sputum samples, four from bronchial aspirates, three from corneal scrapings, and one from paranasal sinus biopsy. Nine *F. oxysporum* isolates were derived from sputum samples, two from bronchial aspirates, two from blood, and one from bronchoalveolar lavage fluids (BALs). Finally, all the *F. proliferatum* samples were from sputum. Corneal scraping and blood culture isolates were obtained from proven invasive fungal infection episodes resulting from deep localization and bloodstream invasion [23]. Otherwise, the respiratory isolates allowed an infection classification according to the EORTC/MSGERC guidelines [23]. These criteria define invasive respiratory infection based on clinical evidence (i.e., a reverse halo sign), patient rick factors (i.e., neutropenia or immunological impairments), and mycological evidence, such as *Fusarium* spp. isolation from any respiratory sample (sputum, bronchial aspirate, or BAL). Since our patients reported at least one clinical sign, one risk factor, and mycological culture isolation, their cases were all categorized as probable invasive respiratory mould infections. Table 2 summarizes MIC ranges, MIC50, and MIC90 of the tested antifungal drugs.

**Figure 3:**
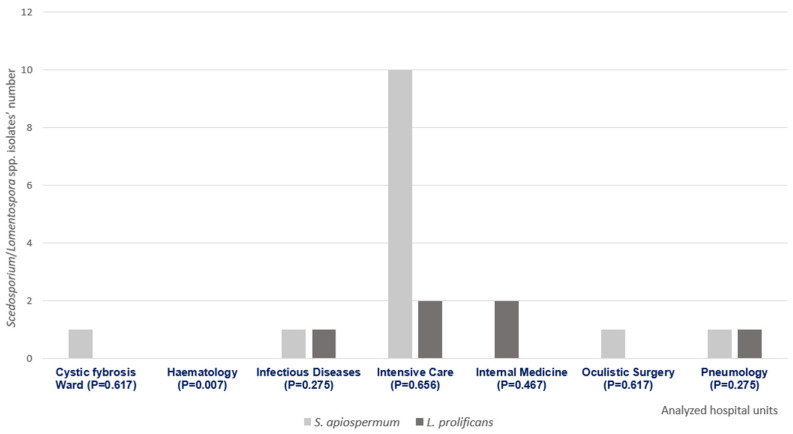
Distribution of *Scedosporium* spp. and *Lomentospora* spp. isolates within the analyzed hospital units. The graph illustrates the different *Fusarium* species’ distributions, also describing the eventual statistical significance (*p* < 0.05) between the identified species and the analyzed hospital ward.

We collected 16 *S. apiospermum* and 4 *L. prolificans*. Figure 3 illustrates their distribution within the involved hospital wards and the corresponding statistical analysis. This analysis illustrates the statistical significance of the absence of *Scedosporium*/*Lomentospora* spp. isolates in the hematology unit.

**Figure 4:**
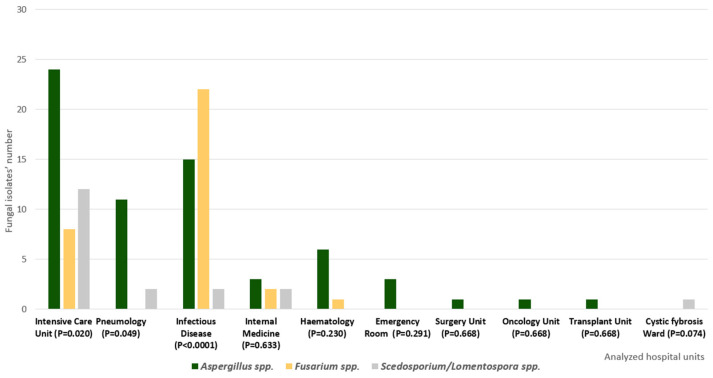
Distribution of *Aspergillus* spp., *Fusarium* spp., and *Scedosporium*/*Lomentospora* spp. isolates within the analyzed hospital units, along with the eventual statistical significance (*p* < 0.05) between the identified genus and the hospital ward. This analysis excluded the oculistic surgery unit, which harboured only corneal scraping isolates.

As regards *S. apiospermum*, we isolated seven strains from BAL, four from bronchial aspirates, four from sputum samples, and one from corneal scrapings. All the *L. prolificans* strains emerged from respiratory samples (two from sputum, one from BAL, and one from bronchial aspirate). The above-mentioned criteria [23] allowed infection classification into a proven case, based on the positive result of corneal scraping, and several probable invasive respiratory infections, based on respiratory sample cultures. Table 3 reports the MIC ranges, MIC90, and MIC50 of the tested antifungal drugs against *Scedosporium* spp. isolates. Finally, Figure 4 illustrates the distribution of *Aspergillus* spp., *Fusarium* spp., and *Scedosporium*/*Lomentospora* spp. within the different hospital units in cases of invasive respiratory infection, along with a corresponding statistical analysis, which emphasizes the significance (*p* < 0.05) of intensive care and infectious disease units.

Table 4 and Table 5 summarize detailed MIC values for *Fusarium proliferatum* and *Lomentospora prolificans*, whose isolate numbers were not sufficient to establish MIC50 or MIC90.

## 3. Discussion

The present manuscript was designed to contribute additional data to the current literature on antifungal susceptibility trends among moulds, emphasizing the epidemiological scenario of Southern Italy and the MIC value distribution. We focused our attention on *Aspergillus* spp., *Fusarium* spp., *Scedosporium apiospermum*, and *Lomentospora prolificans* due to relevant documentation of their intrinsic susceptibility profiles against azoles and/or echinocandins [18,19,20]. Furthermore, all of these fungal pathogens have been included within latest WHO priority list, both for antifungal resistance rates and diffusion capability within critically ill patients [3].

This focus was motivated by the increasing frequency of antifungal resistance reported in filamentous fungi, especially involving *Aspergillus fumigatus* and resistance to triazoles. Several multicentre studies and epidemiological surveillance programmes have documented a progressive rise in triazole-resistant *A. fumigatus* isolates, often associated with prior antifungal exposure or the extensive use of azole compounds in agricultural settings [25]. Considering the first-line role of triazoles in the treatment of invasive aspergillosis, the clinical implications of this phenomenon are substantial.

As a consequence, amphotericin B has been proposed as a potential alternative therapeutic option in geographical areas characterized by a high prevalence of azole resistance [26,27]. In agreement with previous reports, *A. fumigatus* represented the most frequently isolated species among *Aspergillus* spp. in our collection.

Notably, our findings indicated a proportion of *Aspergillus* spp. isolates showing reduced susceptibility to amphotericin B (9.1%). Although amphotericin B has historically retained good activity against *Aspergillus* spp., this observation may suggest the presence of slight changes in local susceptibility patterns when compared to historical epidemiological data. Nevertheless, such findings should be interpreted with caution, as amphotericin B resistance in *Aspergillus* species remains incompletely understood and is considered a multifactorial phenomenon. Importantly, the MIC values recorded in the present study are largely consistent with those previously reported for *Aspergillus* spp. isolates originating from the same geographical area [28,29,30]. In this context, our results may provide a rationale for further investigations, including in vitro combination or synergy studies involving amphotericin B and other antifungal agents, which remain relatively underexplored for *Aspergillus* spp. isolates [28,29,30].

*Fusarium* spp. are extensively known for their elevated MIC values for azoles and amphotericin B, along with a demonstrated intrinsic resistance to echinocandins. Unfortunately, these moulds are often related to negative clinical outcomes due to difficult therapeutic management [31]. However, the genus includes heterogeneous species, and our data enforced this assumption. Specifically, amphotericin B and triazoles (voriconazole, posaconazole) revealed high MIC values for all the collected *F. solani* strains. On the other hand, *F. oxysporum* and *F. proliferatum* showed lower MIC values for the same antifungal drugs. Scientific and clinical case reports have highlighted the critical conditions related to *L. prolificans* and *S. apiospermum* infections [32]. These species frequently exhibit antifungal resistance, and our data confirmed the same criticism, revealing high MIC values for both of the above-mentioned species in the case of echinocandins, azoles, and amphotericin B.

Our study certainly has several limitations. Unfortunately, invasive fungal infections caused by filamentous fungi are significantly underestimated due to limited sensitivity rates within diagnostic protocols. Moreover *Aspergillus* spp., *Scedosporium* spp., and *Scedosporium*/*Lomentospora* spp. isolates are difficult-to-manipulate pathogens in most laboratory routines. They are often related to intense conidia aerosolization, technical expertise requirements, and dedicated specialized personnel. Consequently, culture isolation, identification, and antifungal susceptibility testing are highly complicated to perform. According to these assumptions, our study included a few isolates for each analyzed species. More studies with higher sample sizes will be necessary to further investigate in vitro mould susceptibility trends. Furthermore, molecular characterization of certain resistance mechanisms may clarify specific antifungal resistance trends.

Finally, susceptibility testing profiles, including ultimate antifungal drugs (e.g., isavuconazole and rezafungin), may be ideal to enforce epidemiological and clinical considerations.

In our opinion, our collected data highlight the necessity of continuously updating antifungal susceptibility trends for filamentous fungi. Regrettably, in vitro methodologies have not been sufficiently optimized for this purpose, and extended investigations should be conducted to achieve their official standardization in mould testing.

## 4. Materials and Methods

This study included a four-year (2021–2024) epidemiological analysis of all filamentous fungi isolated from clinical samples of patients, recovered at the University Hospital Policlinico, Catania (Italy). Specifically, the analysis included moulds from respiratory samples (bronchoalveolar lavage fluid, sputum, bronchial aspirate) and corneal scrapings. According to the routinary laboratory procedure, we inoculated these biological samples into Sabouraud Dextrose Agar with 2% of glucose, incubating the plates at 30 °C for 7 days. The MALDI Biotyper^®^ Sirius System mass spectrometry technology (Bruker, Billerica, MA, USA) identified the eventual filamentous fungi colonies. Specifically, fragments from grown colonies underwent a chemical extraction step involving ethanol, formic acid, and acetonitrile. Extracted fungal material subsequently went through centrifugation processes, collecting a supernatant. An aliquot of 1 µL was inoculated into the MALDI plate and covered with 1 µL of supplementary formic acid and α-cyano-4-hydroxycinnamic acid (HCCA) matrix. Regarding fungal species excluded from the MALDI identification panel, we identified *L. prolificans* based on microscopic and macroscopic morphological features, supporting our evidence with the absence of any growth in Sabouraud Dextrose agar with 2% of glucose and cycloheximide subcultures.

In vitro antifungal susceptibility was defined using Sensititre Yeast-One (Thermo Fisher Scientific, Waltham, MA, USA), with interpretation following the manufacturer’s instructions (https://documents.thermofisher.com/TFS-Assets/MBD/Package-Inserts/018-_PIYSTIVD-US-V3.2_CID9833.pdf, accessed on 4 January 2021). The inoculated plates were incubated at 37 °C for 48 h before verifying the antifungal susceptibility profile. This method has been clinically validated for yeast susceptibility testing, but it has also been extensively investigated for *Aspergillus* spp., *Fusarium* spp., and *Scedosporium* spp. in specific laboratory-validated contexts [33,34,35,36]. Additionally, previously published studies have highlighted the possibility of applying the Sensititre method to diagnostic workflows due to its elevated agreement with standardized broth microdilution [37,38,39,40,41].

We categorized the gathered MIC values according to filamentous fungi CLSI guidelines, reporting some clinical breakpoints (CBPs) for *Aspergillus* spp. isolates [15]. MICs higher than epidemiological cut-off (E-COFF) values were attributed to hypothetically resistant isolates in the absence of clinical breakpoints. For echinocandins tested against *Aspergillus* spp., minimum effective concentrations (MECs) were determined based on the presence of abnormal, short, and highly branched hyphal growth, rather than conventional MIC endpoints. All the resistance MIC values or the MIC values higher than the E-COFF were confirmed through the CLSI standardized broth microdilution method. In the cases of *Fusarium* spp., *S. apiospermum*, and *L. prolificans*, we did not classify the reported MIC values due to the absence of CBP and E-COFF.

Moreover, we reported MIC ranges for all the analyzed species, while MIC50 and MIC90 were calculated only for species with isolate numbers equal to or higher than 10 (*Aspergillus fumigatus*, *Aspergillus niger*, *Aspergillus flavus*, *Aspergillus terreus*, *Fusarium solani*, *Fusarium oxysporum*, and *Scedosporium apiospermum*). We followed these minimum conditions to guarantee statistically balanced isolate populations. The occurrence of trailing growth was mainly observed with azole antifungals and led to MICs, defined as the lowest drug concentration causing a significant reduction in growth compared to the drug-free growth control.

We performed a statistical analysis of *Aspergillus* spp., *Fusarium* spp., and *Scedosporium/Lomentospora* spp. within the included hospital units, using MedCalc Statistical Software version 17.9.2 (MedCalc Software bvba, Ostend, Belgium; http://www.medcalc.org; 2017; accessed on 9 January 2026) and reporting the corresponding *p* values. Specifically, χ^2^ and Fisher’s exact tests were applied to establish the categorical variables as percentages. The same statistical analysis finally assessed the distribution of *Aspergillus* spp., *Fusarium* spp., and *Scedosporium*/*Lomentospora* spp. genera within the different hospital units in cases of invasive respiratory infection.

## 5. Conclusions

The present study confirmed a concerning increase in filamentous fungi resistance percentages, particularly highlighting reduced susceptibility in *A. fumigatus*, *F. solani*, and *L. prolificans*. Our results emphasize the importance of enforcing epidemiological surveys, optimizing susceptibility testing, and performing precise species identification in the laboratory diagnosis of invasive fungal infection.

## Figures and Tables

**Table 1:** Antifungal susceptibilities of *Aspergillus* spp. to antifungal agents.

Mould Species and Antifungal Agent	Minimum Inhibitory Concentration			In Vitro Susceptibility; No. (%)	
	Range (mg/L)	MIC50	MIC90	S	R
***A. fumigatus* (28)**					
Amphotericin B *	1–4	2	4	25 (89.3%)	3 (10.7%)
Caspofungin *	0.03–0.06	0.06	0.06	28 (100%)	0
Posaconazole	0.03–1	0.06	0.125	NA	NA
Fluconazole	256	256	256	NA	NA
Itraconazole *	0.12–0.5	0.12	0.125	28 (100%)	0
Voriconazole **	0.125–1	0.5	1	28 (100%)	0
***A. flavus* (16)**					
Amphotericin B *	0.5–4	1	4	16 (100%)	0
Caspofungin *	0.03–0.06	0.06	0.06	16 (100%)	0
Posaconazole *	0.125–1	0.125	1	16 (100%)	0
Fluconazole	256	256	256	NA	NA
Itraconazole *	0.12–0.25	0.25	0.25	16 (100%)	0
Voriconazole *	0.5–1	0.5	1	16 (100%)	0
***A. niger* (11)**					
Amphotericin B *	0.25–1	0.5	1	11 (100%)	0
Caspofungin *	0.008–0.03	0.03	0.03	11 (100%)	0
Posaconazole *	0.03–0.5	0.5	0.5	11 (100%)	0
Fluconazole	256	256	256	NA	NA
Itraconazole *	0.12–1	1	1	11 (100%)	0
Voriconazole *	0.25–1	1	1	11 (100%)	0
***A. terreus* (10)**					
Amphotericin B *	2–8	4	8	7 (70%)	3 (30%)
Caspofungin *	0.015–0.25	0.25	0.25	10 (100%)	0
Posaconazole *	0.06–0.25	0.125	8	10 (100%)	0
Fluconazole	256	256	256	NA	NA
Itraconazole *	0.06–16	0.5	16	9 (90%)	1 (10%)
Voriconazole *	0.125–4	0.25	4	9 (90%)	1 (10%)

Abbreviations: S = susceptible, calculated considering MIC values lower than the epidemiological cut-off (*), and equal to or lower than the clinical breakpoint (**); R = resistant, classified considering MIC values higher than the epidemiological cut-off (*), and equal to or higher than the clinical breakpoint (**); NA = not applicable due to the absence of both clinical breakpoints and epidemiological cut-off values.

**Table 2:** Antifungal susceptibilities of *Fusarium* spp. to antifungal agents.

Mould Species and Antifungal Agent	Minimum Inhibitory Concentration			In Vitro Susceptibility	
	Range (mg/L)	MIC50	MIC90	S	R
***Fusarium solani* (19)**					
Amphotericin B	2–4	4	4	NA	NA
Caspofungin	8	8	8	NA	NA
Posaconazole	8	8	8	NA	NA
Fluconazole	128–256	256	256	NA	NA
Itraconazole	6–8	8	8	NA	NA
Voriconazole	8	8	8	NA	NA
***Fusarium oxysporum* (14)**					
Amphotericin B	0.25–1	1	1	NA	NA
Caspofungin	8	8	8	NA	NA
Posaconazole	0.5–8	8	8	NA	NA
Fluconazole	8–16	8	16	NA	NA
Itraconazole	2–8	4	8	NA	NA
Voriconazole	0.25–8	2	8	NA	NA
***Fusarium proliferatum* (3)**					
Amphotericin B	0.25–2	NA	NA	NA	NA
Caspofungin	8	NA	NA	NA	NA
Posaconazole	0.5–4	NA	NA	NA	NA
Fluconazole	8	NA	NA	NA	NA
Itraconazole	8	NA	NA	NA	NA
Voriconazole	0.25–2	NA	NA	NA	NA

NA = Not applicable due to insufficient isolate numbers (MIC50 and MIC90 calculations require a minimum of 10 isolates per species) or absence of both clinical breakpoints and epidemiological cut-off values.

**Table 3:** Antifungal susceptibilities of *Scedosporium apiospermum* and *Lomentospora prolificans* to antifungal agents.

Mould Species and Antifungal Agent	Minimum Inhibitory Concentration			Minimum Inhibitory Concentration	
	Range (mg/L)	MIC50	MIC90	S	R
***S. apiospermum* (16)**					
Amphotericin B	4–8	4	4	NA	NA
Caspofungin	4–8	8	8	NA	NA
Posaconazole	0.25–16	0.25	0.25	NA	NA
Fluconazole	8–32	16	16	NA	NA
Itraconazole	0.125–8	0.25	0.25	NA	NA
Voriconazole	0.25–8	8	8	NA	NA
***L. prolificans* (4)**					
Amphotericin B	8	NA	NA	NA	NA
Caspofungin	16	NA	NA	NA	NA
Posaconazole	16	NA	NA	NA	NA
Fluconazole	16	NA	NA	NA	NA
Itraconazole	8	NA	NA	NA	NA
Voriconazole	8	NA	NA	NA	NA

NA = Not applicable due to insufficient isolate numbers (MIC50 and MIC90 calculations require a minimum of 10 isolates per species) or absence of both clinical breakpoints and epidemiological cut-off values.

**Table 4:** Detailed MIC values for *Fusarium proliferatum* isolates.

Species	AB (mg/L)	CAS (mg/L)	PZ (mg/L)	FZ (mg/L)	IZ (mg/L)	VOR (mg/L)
*Fusarium proliferatum*	0.25	8	0.5	8	8	0.25
*Fusarium proliferatum*	2	8	2	8	8	0.5
*Fusarium proliferatum*	0.5	8	4	8	8	2

Abbreviations: AB = amphotericin B; CAS = caspofungin; PZ = posaconazole; FZ = fluconazole; IZ = itraconazole; VOR = voriconazole.

**Table 5:** Detailed MIC values for *Lomentospora prolificans* isolates.

Species	AB (mg/L)	CAS (mg/L)	PZ (mg/L)	FZ (mg/L)	IZ (mg/L)	VOR (mg/L)	MF (mg/L)
*Lomentospora prolificans*	0.25	8	0.5	8	8	0.25	8
*Lomentospora prolificans*	2	8	2	8	8	0.5	8
*Lomentospora prolificans*	0.5	8	4	8	8	2	8

Abbreviations: AB = amphotericin B; CAS = caspofungin; PZ = posaconazole; FZ = fluconazole; IZ = itraconazole; VOR = voriconazole.

## Data Availability

All the gathered results have been included within the present manuscript.
